# High-Density Genetic Linkage Map Construction and Quantitative Trait Locus Mapping for Hawthorn (*Crataegus pinnatifida* Bunge)

**DOI:** 10.1038/s41598-017-05756-5

**Published:** 2017-07-14

**Authors:** Yuhui Zhao, Kai Su, Gang Wang, Liping Zhang, Jijun Zhang, Junpeng Li, Yinshan Guo

**Affiliations:** 1 College of Horticulture, Shenyang Agricultural University, Shenyang, P.R. China; 2 College of Horticulture Sciences & Technology, Hebei Normal University of Science & Technology, Qinhuangdao, China

## Abstract

Genetic linkage maps are an important tool in genetic and genomic research. In this study, two hawthorn cultivars, Qiujinxing and Damianqiu, and 107 progenies from a cross between them were used for constructing a high-density genetic linkage map using the 2b-restriction site-associated DNA (2b-RAD) sequencing method, as well as for mapping quantitative trait loci (QTL) for flavonoid content. In total, 206,411,693 single-end reads were obtained, with an average sequencing depth of 57× in the parents and 23× in the progeny. After quality trimming, 117,896 high-quality 2b-RAD tags were retained, of which 42,279 were polymorphic; of these, 12,951 markers were used for constructing the genetic linkage map. The map contained 17 linkage groups and 3,894 markers, with a total map length of 1,551.97 cM and an average marker interval of 0.40 cM. QTL mapping identified 21 QTLs associated with flavonoid content in 10 linkage groups, which explained 16.30–59.00% of the variance. This is the first high-density linkage map for hawthorn, which will serve as a basis for fine-scale QTL mapping and marker-assisted selection of important traits in hawthorn germplasm and will facilitate chromosome assignment for hawthorn whole-genome assemblies in the future.

## Introduction

Hawthorn (*Crataegus pinnatifida* Bunge) belongs to the family Rosaceae and is a widespread fruit tree in China. The fruits are used for food and medicinal purposes. The leaves, fruits, roots, and twigs of hawthorn contain many nutrients, including proteins, fats, dietary fibre, vitamins, flavones, and many minerals. Many studies have focused on hawthorn flavones^1–6^, which are the primary bioactive components^7^. Flavones are polyphenol secondary metabolites that have low molecular weight and are common in plants; they are produced in response to environmental stress and play a role in defence against predators and pathogens^8^. Flavones have antioxidant and anticancer properties and can scavenge free radicals^9^, so their properties have been widely researched for use in agriculture, the chemical industry and medicine. Flavone production is under genetic regulation, but the regulation of flavones in hawthorn has been little studied.

Genetic linkage maps, particularly high-density maps, are one of the most valuable tools for high-throughput selection of superior traits from plant and animal germplasms. Our lab published the first hawthorn genetic linkage map that was constructed using sequence-related amplified polymorphism markers^10^, but its application value was limited because it included relatively few markers with long marker intervals.

As high-throughput technology and next-generation sequencing (NGS) methods have been developed, many can now quickly genotype thousands of markers in a single step^11^. Restriction-site associated DNA sequencing (RAD-seq)^12, 13^, specific length amplified fragment (SLAF) sequencing^14^, and genotyping by sequencing (GBS, or NGS)^15^ are powerful tools for constructing high-density genetic linkage maps. For example, Pfender *et al*.^16^, Chutimanitsakun *et al*.^17^, and Wang *et al*.^18^ used RAD-seq to construct high-density genetic linkage maps for ryegrass, barley, and grape, respectively. Poland *et al*.^15^ constructed high-density genetic linkage maps for barley and wheat, and Zhang *et al*.^19^ constructed a map for jujube based on GBS technology. Several other studies have used SLAF to construct high-density genetic linkage maps for soybean^20^, sesame^21^, and *Salvia miltiorrhiza* Bunge^22^.

Type IIB endonuclease RAD (2b-RAD) uses restriction enzymes such as *Bsa*XI or *Alf*I (both insensitive to methylation) to produce uniform tags. This is a simple and flexible method for genome-wide genotyping^23^.

In this study, our aim was to construct a high-density genetic linkage map for hawthorn using the 2b-RAD method and then conduct fine-scale QTL mapping for flavonoid content in hawthorn leaves. This linkage map will be a powerful tool for research involving both fine-scale QTL mapping and marker-assisted selection of important economic traits of hawthorn germplasm. It will facilitate chromosome assignment for a future whole-genome assembly.

## Results

### 2b-RAD sequencing and markers selection

**Figure 1:**
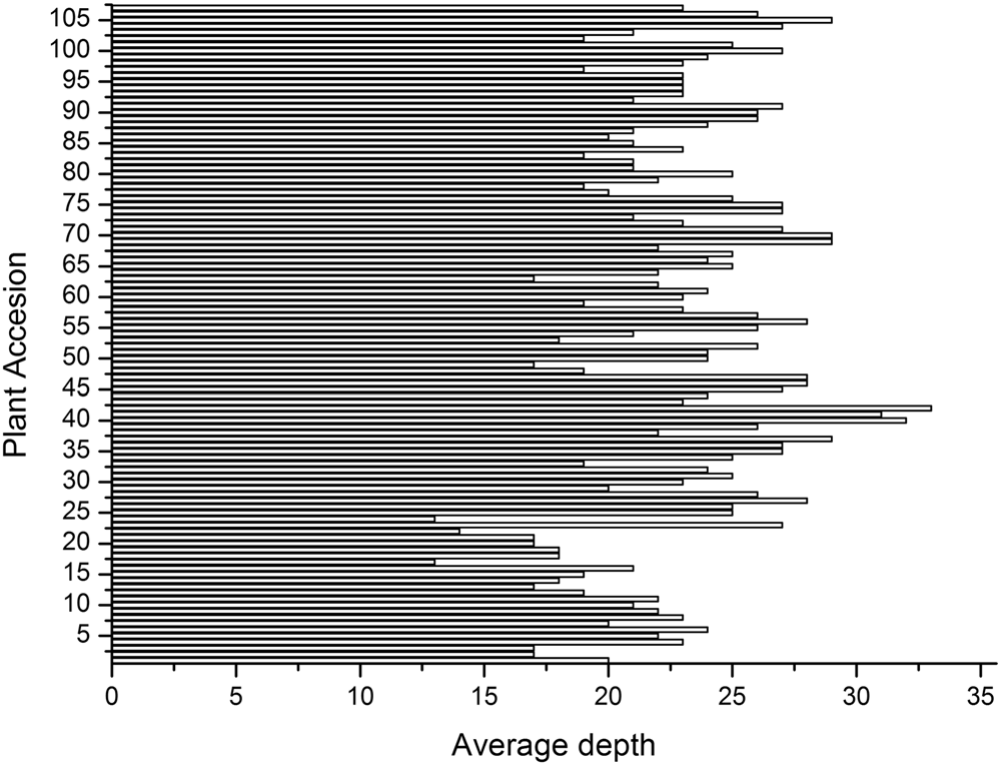
Sequencing depth of 107 progeny. The x-axes indicate average depth and the y-axes indicate individual plant accessions.

**Figure 2:**
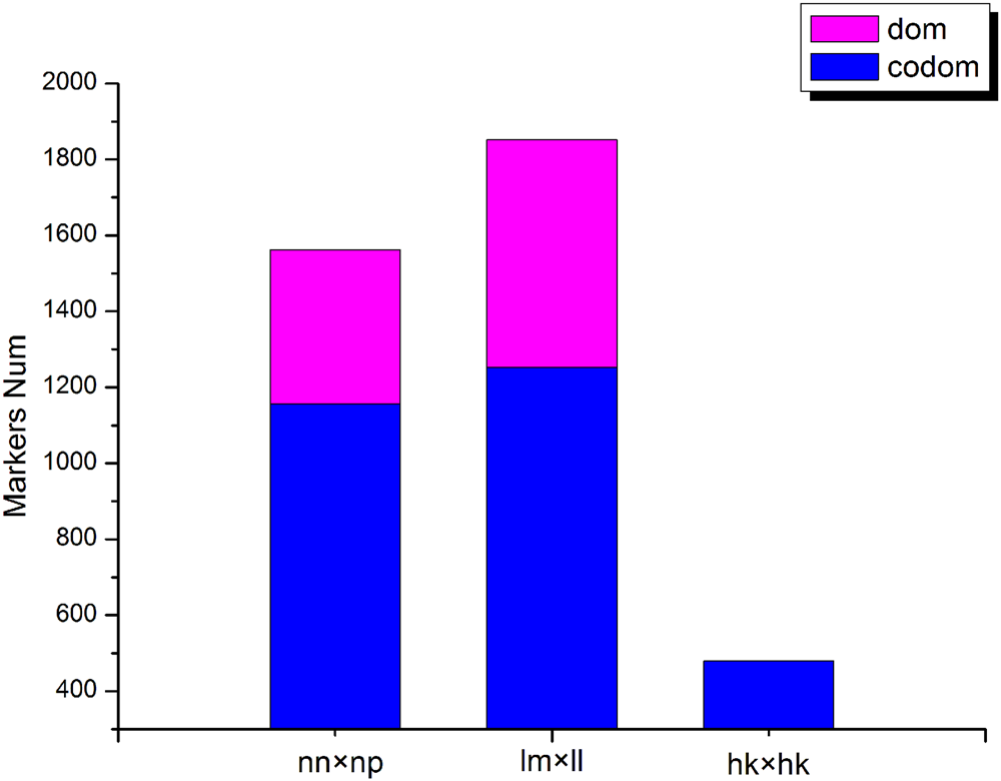
Number of markers for each segregation pattern. The x-axes means marker type and y-axes means marker number.

The Hiseq2500 v2 platform was used to conduct single-end sequencing of 2b-RAD libraries for the parents and 107 progenies. A total of 526,949,839 reads were recovered, including 11,265,798 reads from the seed parent, 12,977,353 reads from the pollen parent, and 502,706,688 reads from the progeny. The sequencing depth was 62× for the seed parent, 52× for the pollen parent, and 23× for the 107 progenies. The sequencing depth detail of the 107 progenies is shown in Fig. 1. After low-quality reads were trimmed, 82.39% of reads from the seed parent, 82.67% of reads from the pollen parent, and 84.50% of reads from the progeny were retained for analysis (Table 1). Using SOAP software^24^, 117,896 unique tags were generated, including 13,937 dominant tags and 103,959 codominant tags. RAD typing v1.0 software^25^ was used to genotype the reads, with 47,923 SNPs that were polymorphic markers, including 9,237 dominant markers and 38,686 codominant markers. A Mendelian fit test and genotyping percentage were used to trim SNP markers for 12,951 markers that fit Mendelian ratios (P ≥ 0.05) and possessed a high genotyping rate (the available genotype was found in over 80% of progeny), and the types of all these markers are shown in Fig. 2. These markers were used for constructing parental maps. Finally, 6,390 markers were used for constructing a seed parent map and 7,384 markers were used for constructing a pollen parent map. In total, 823 markers were shared by the two parents (Table 2).

**Table 1:** Summary of data filtering of Hawthorn.

	Raw-reads	High-quality reads	Sequencing depth
Male	11,265,798	9,313,823 (82.67%)	52
Female	12,977,353	10,691,957 (82.39%)	62
Progeny	4,698,193	3,970,014 (84.50%)	23

**Table 2:** Markers selected for linkage mapping.

	Number
Markers detected in parents	117896
Markers polymorphic and with high genotype percentage	47923
Markers used for linkage mapping after Mende-lian ratio trimming	12951
Markers used for seed parent linkage mapping	6390
Markers used for pollen parent linkage mapping	7384

### Number of homozygous and heterozygous SNPs and population structure analysis

**Figure 3:**
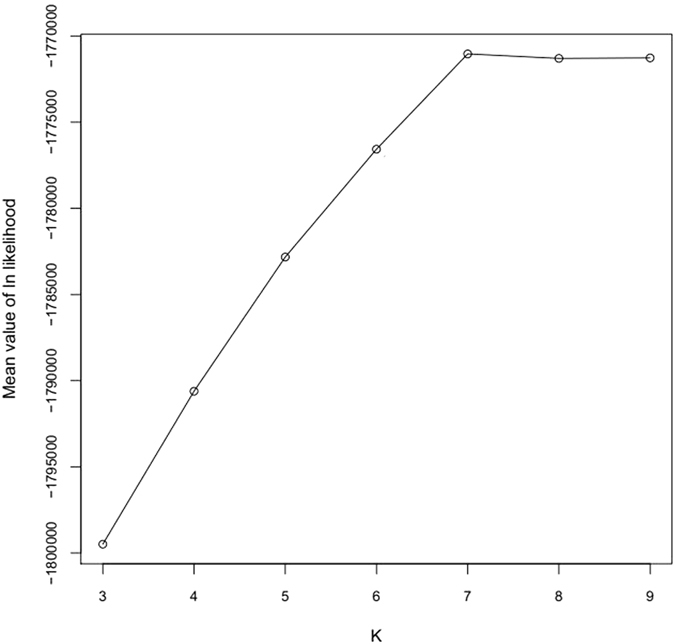
Optimal K selection. The x-axes means K value and y-axes was mean value of ln likelihood respond to K value.

**Figure 4:**
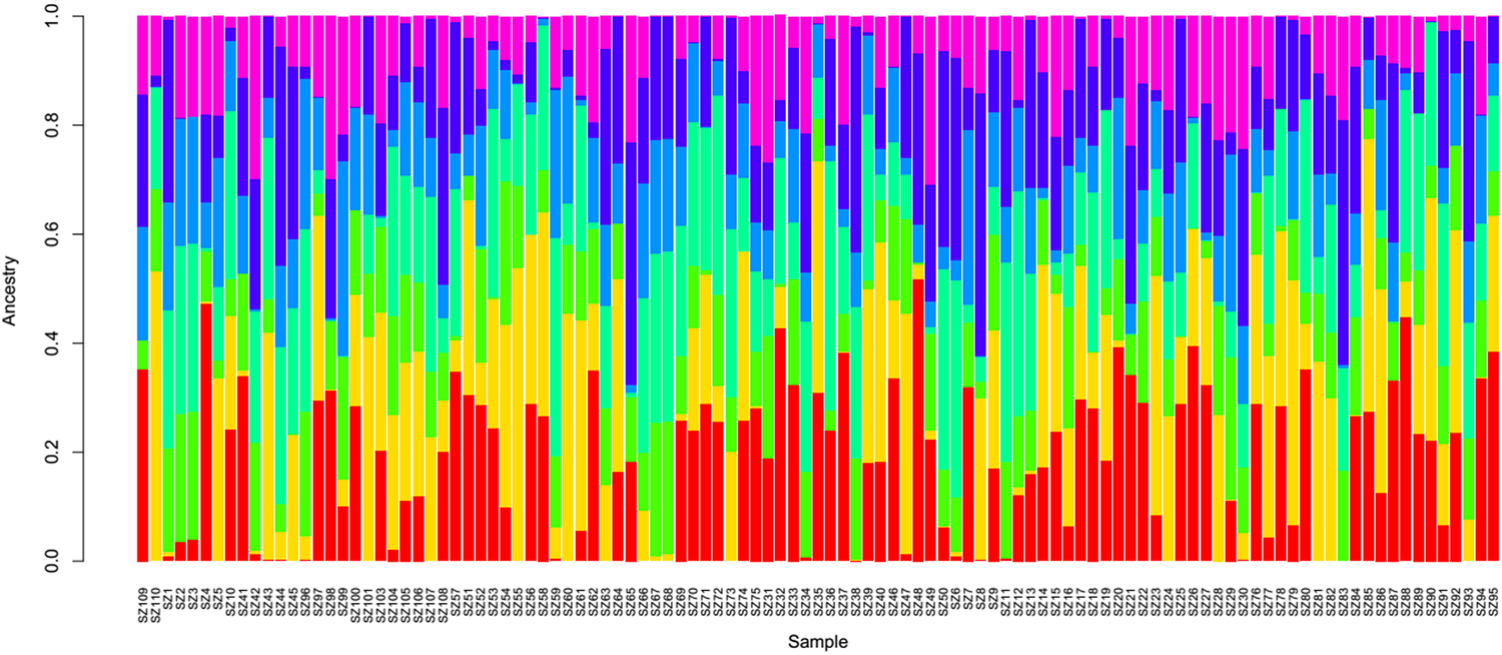
Population structure. The x-axes indicate individual plant accessions and y-axes means ancestries of each individual plant accessions.

The number of homozygous and heterozygous SNPs are listed in Additional File A1 and Additional File A2. Plant material (SZ7) had the most homozygous SNPs markers and plant material (SZ110) had the most heterozygous SNPs markers. SZ7 has the highest Ho/He rate. The population structure was calculated using Structure software based on the SNPs data. Parameter K was settled from 3 to 9. The optimal K value was 7 and is shown in Fig. 3. Based on the optimal K value, structural analysis was then conducted using Structure software, and the result was shown in Fig. 4. In all, 108 progenies and 2 parents were clustered into 7 subpopulations.

### Construction of high-density linkage map

The high-density parental linkage maps were first constructed using Joinmap4.1 software^26^ (logarithm of odds (LOD) ≥5) to map 1,890 markers for the seed parent and 2,149 markers for the pollen parent. The parental maps contained 17 linkage groups, which were consistent with the haploid chromosome number of hawthorn^10^.

**Figure 5:**
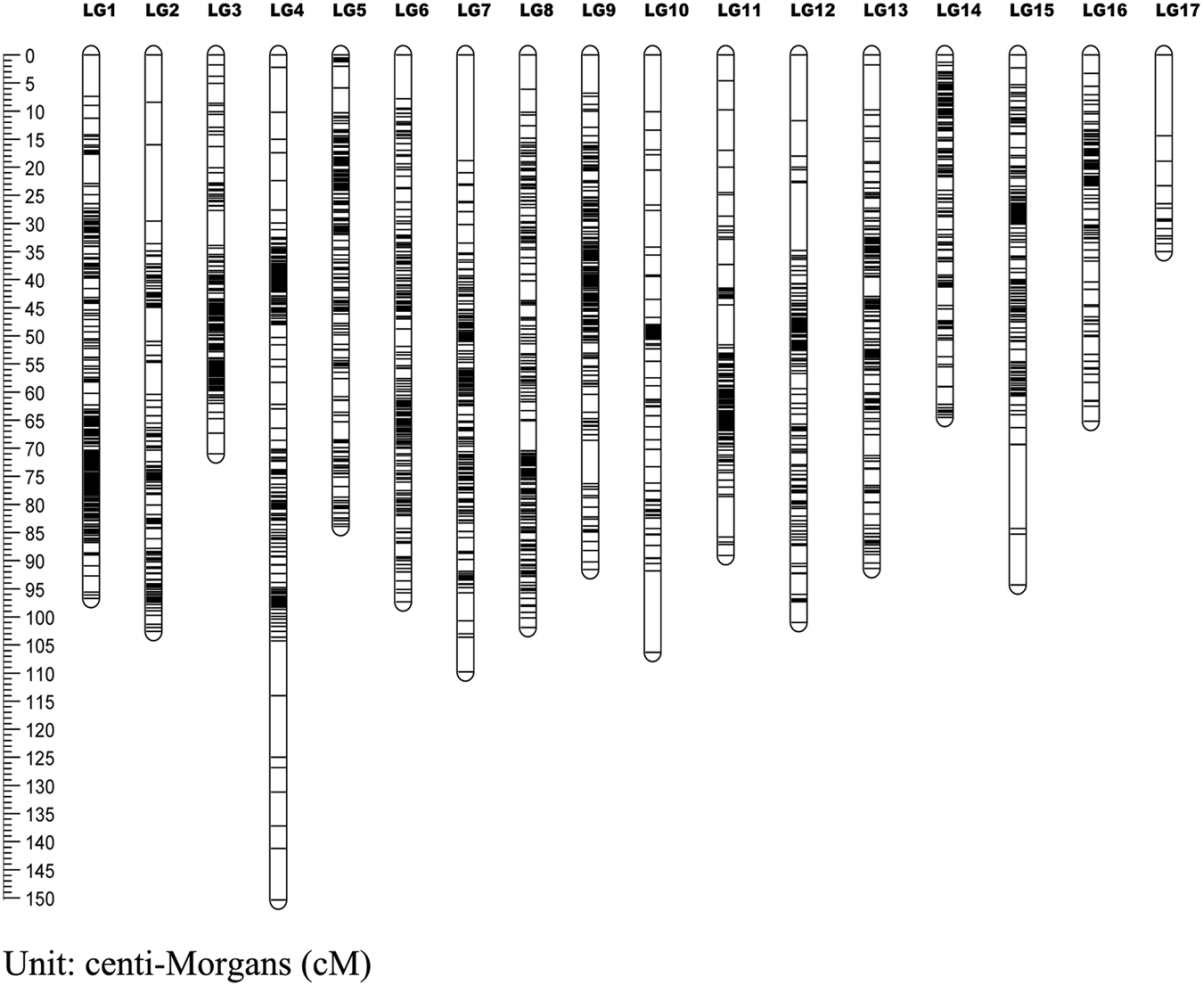
Genetic lengths and marker distribution in 17 linkage groups of consensus linkage map. A black bar means a 2b-RAD marker. The left scale plate imeans genetic distance (centiMorgan as unit).

For the seed parent linkage map, 1,890 markers were mapped to 1,780 distinct positions. The total linkage map length was 1,266.81 cM, the longest linkage group, LG7, was 107.93 cM, the shortest linkage group, LG17, was 34.43 cM, and the average linkage group length was 74.52 cM. LG6, which contained 117 markers, had the highest number of markers, while LG17 contained the lowest number of markers at 15; the average marker number for these 17 linkage groups was 111. The longest average marker interval of 2.46 cM was found in LG17, the shortest average marker interval of 0.38 cM was found in LG6, and the average marker interval of these 17 linkage groups was 0.68 cM. The percentage of ‘Gap ≤5′ was used to reflect the linkage level between adjacent markers in the same linkage group. In the seed parent map, LG15 had the lowest percentage (82.05), while LG14 had the highest percentage (100.00) (Table 3 and Fig. 5).

**Table 3:** Summary of sex-specific linkage maps of Hawthorn.

LG	Seed parent map					Pollen parent map				
	Mapped markers	Distinct positions	Genetic length (cM)	Marker interval (cM)	Gaps ≤5 (Max gap)	Mapped markers	Distinct positions	Genetic length (cM)	Marker interval (cM)	Gaps ≤5 (Max gap)
1	161	156	84.42	0.53	97.42%	172	169	91.68	0.54	98.21%
2	99	99	99.57	1.02	94.90%	217	121	59.62	0.28	98.33%
3	83	80	48.18	0.59	97.47%	224	220	62.70	0.28	99.54%
4	99	99	92.26	0.94	94.90%	183	178	87.38	0.48	97.18%
5	131	131	61.09	0.47	99.23%	151	148	79.37	0.53	98.64%
6	171	150	64.06	0.38	99.33%	116	113	94.42	0.82	98.21%
7	143	142	107.93	0.76	97.16%	116	116	84.22	0.73	97.39%
8	116	114	95.59	0.83	97.35%	145	144	97.86	0.68	97.90%
9	107	106	62.81	0.59	98.10%	139	138	73.46	0.53	97.81%
10	167	107	89.16	0.54	96.23%	67	64	80.06	1.21	95.24%
11	101	98	71.45	0.71	97.94%	110	103	56.68	0.52	98.04%
12	94	89	81.02	0.87	95.45%	114	113	97.26	0.86	97.32%
13	124	121	66.67	0.54	98.33%	91	89	71.39	0.79	98.86%
14	145	141	62.49	0.43	100.00%	68	67	55.29	0.83	95.45%
15	40	40	86.01	2.21	82.05%	163	160	86.51	0.53	98.74%
16	94	93	59.69	0.64	98.91%	61	61	64.90	1.08	96.67%
17	15	14	34.43	2.46	92.33%	12	11	14.23	1.29	90.00%
Total	1890	1780	1266.81	0.68	96.30%	2149	2015	1257.02	0.59	97.27%

**Figure 6:**
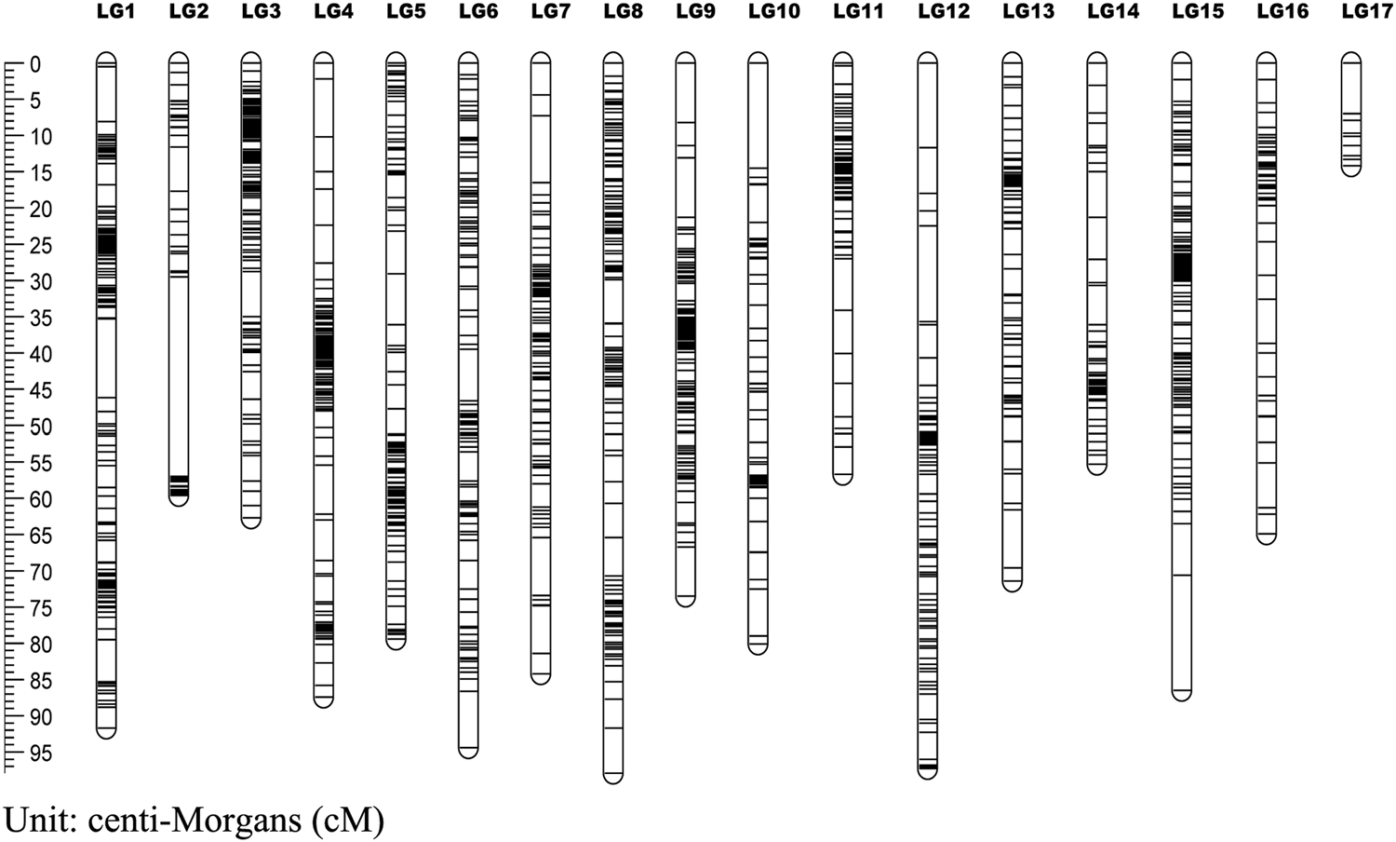
Genetic lengths and marker distribution in 17 linkage groups of pollen parent linkage map. A black bar means a 2b-RAD marker. The left scale plate imeans genetic distance (centiMorgan as unit).

For the pollen parent linkage map, 2,149 markers were mapped to 2,015 distinct positions. The total linkage map length was 1257.02 cM, the longest linkage group, LG8, was 97.86 cM, the shortest linkage group, LG17, was 14.23 cM, and the average linkage group length was 73.94 cM. LG3, which contained 224 markers, had the highest number of markers, while LG17 contained the lowest number of markers at 12; the average marker number for these 17 linkage groups was 126. The longest average marker interval of 1.29 cM was found in LG17, the shortest average marker interval of 0.28 cM was found in LG2, and the average marker interval of these 17 linkage groups was 0.59 cM. In the pollen parent map, LG17 had a lowest percentage of ‘Gap ≤5′ (90.00), while LG3 had the highest percentage (99.54) (Table 3 and Fig. 6).

**Figure 7:**
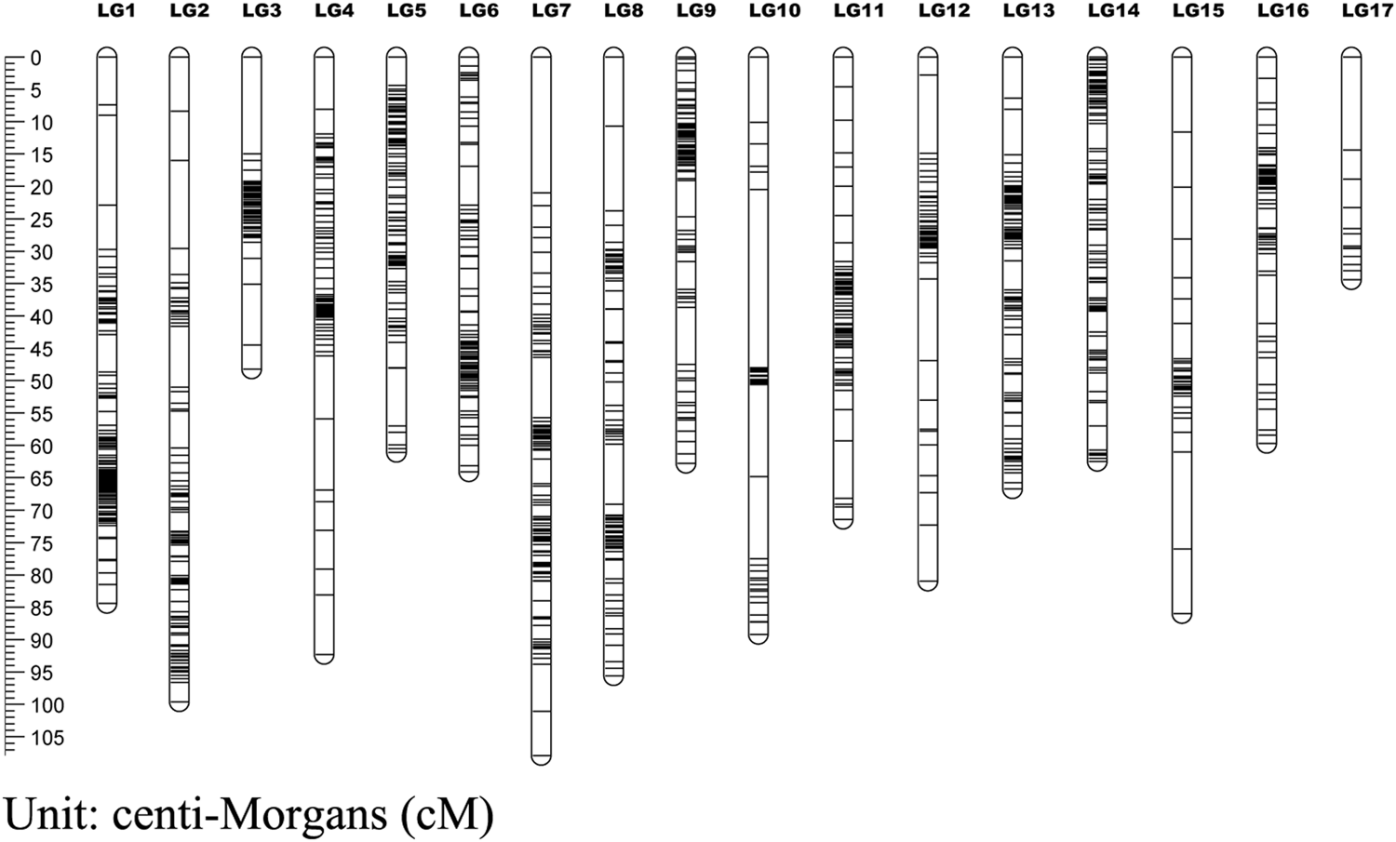
Genetic lengths and marker distribution in 17 linkage groups of seed parent linkage map. A black bar means a 2b-RAD marker. The left scale plate imeans genetic distance (centiMorgan as unit).

The hawthorn consensus linkage map was constructed by integrating the two parental maps based on 823 shared markers. It contained 3,894 markers that were mapped to 3,296 distinct positions. The total linkage map length was 1,551.97 cM, the longest linkage group was LG4 (150.37 cM), the shortest linkage group was LG17 (35.03 cM), and the average linkage group length was 91.29 cM. LG1 contained the highest number of markers at 327, LG17 contained the lowest number of markers at 16, and the average number of markers for these 17 linkage groups was 229. The longest average marker interval of 2.34 cM was found in LG17, the shortest average marker interval of 0.30 cM was found in LG1, and the average marker interval of these 17 linkage groups was 0.40 cM. In the consensus map, LG17 had the lowest percentage of ‘Gap ≤5′ (92.86), while LG5 had the highest percentage (100.00) (Table 4 and Fig. 7).

**Table 4:** Summary of the consensus linkage map of Hawthorn.

LG	Consensus map							
	Mapped markers	Female-specific makers	Male-specific markers	Shared makers	Distinct positions	Genetic length (cM)	Marker interval (cM)	Gaps ≤5 (Max gap)
1	327	128	157	42	297	96.69	0.30	99.32%
2	312	87	213	12	174	102.63	0.33	97.11%
3	295	47	209	39	243	70.99	0.24	99.59%
4	279	92	179	8	248	150.37	0.54	97.17%
5	275	104	134	37	244	83.93	0.31	100.00%
6	271	146	89	36	217	97.26	0.36	99.54%
7	251	123	95	33	239	109.84	0.44	98.74%
8	244	99	100	45	228	101.92	0.42	99.12%
9	239	81	130	28	226	91.62	0.38	99.11%
10	218	150	30	38	121	106.28	0.49	96.67%
11	208	93	101	14	172	89.07	0.43	97.66%
12	207	87	113	7	178	100.97	0.49	98.31%
13	205	104	39	62	191	91.35	0.45	99.47%
14	200	103	51	46	175	64.53	0.32	100.00%
15	198	34	158	6	189	94.26	0.48	98.94%
16	149	81	54	14	139	65.23	0.44	100.00%
17	16	3	0	13	15	35.03	2.34	92.86%
Total	3894	1562	1852	480	3296	1551.97	0.40	98.45%

### QTL mapping analysis of hawthorn flavonoid content

According to the analysis of five flavonoid component contents in 2014 and 2015, vitexin-rhamnoside was the major component followed by hyperoside, rutin, vitexin and quercetin. The concentrations of vitexin-rhamnoside were 0.00–0.849% and 0.00–0.825% in 2014 and 2015, respectively; the concentrations of hyperoside were 0.00–0.327% and 0.00–0.204% in 2014 and 2015, respectively; the concentrations of rutin were 0.00–0.037% and 0.00–0.078% in 2014 and 2015, respectively; the concentrations of vitexin were 0.00–0.038% and 0.00–0.039% in 2014 and 2015, respectively; and the concentrations of quercetin were 0.00–0.003% and 0.00–0.010% in 2014 and 2015, respectively(Additional File A4).

**Figure 8:**
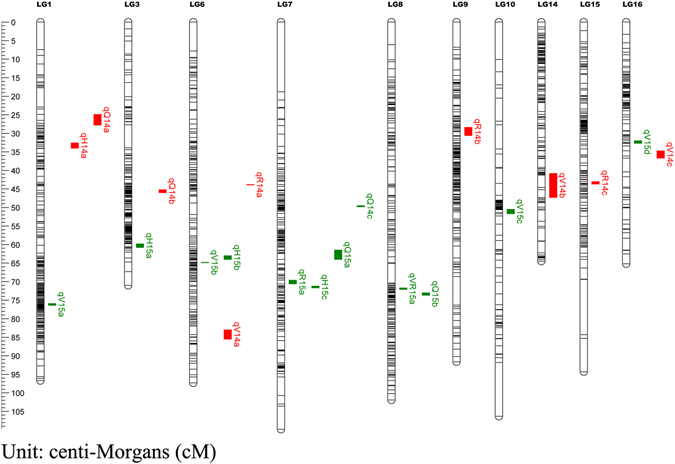
Genomic QTL distribution on 10 different chromosomes.

QTL mapping was conducted for hawthorn leaf flavonoid content, which was measured in 2014 and 2015. In total, 21 QTLs located in 10 linkage groups affected the flavonoid content (Table 5 and Fig. 8).

**Table 5:** All QTLs for 5 flavonoid content-related traits. PVE: phenotypic variance explained.

Trait	Year	QTL name	Marker	LG	Position (cM)	LOD	LOD Threshold		PVE (%)
							Genome-wide	Group-wide	
vitexin-rhamnoside	2015	qVR15a	f1467	8	71.86	3.80	5.1	3.7	17.70
Vitexin	2014	qV14a	m2544	6	84.32	4.24	5.0	3.5	19.50
		qV14b	h528-f1938	14	44.30	3.47	5.0	3.2	16.30
		qV14c	m2175-h534	16	35.68	17.40	5.0	3.2	59.00
	2015	qV15a	df236	1	76.04	3.76	5.1	3.5	17.50
		qV15b	f1741	6	64.81	4.16	5.1	3.5	19.20
		qV15c	m2301	10	51.42	3.58	5.1	3.5	16.70
		qV15d	f1181	16	32.65	14.59	5.1	3.3	52.60
Rutin	2014	qR14a	m2129	6	43.81	4.12	5.0	3.5	19.00
		qR14b	h62	9	29.57	3.74	5.0	3.4	17.40
		qR14c	m209	15	43.53	4.70	5.0	3.6	21.40
	2015	qR15a	h327	7	69.63	5.62	5.0	4.6	25.00
Hyperoside	2014	qH14a	f28	1	33.51	3.71	5.3	3.6	17.30
	2015	qH15a	m1834	3	60.45	3.94	5.3	3.6	18.20
		qH15b	f1703	6	63.89	4.43	5.3	3.8	20.30
		qH15c	h2	7	71.42	4.63	5.3	3.8	21.10
Quercetin	2014	qQ14a	m989	1	26.50	4.81	5.2	3.9	21.80
		qQ14b	h336	3	45.84	4.76	5.2	4.2	21.60
		qQ14c	m279	7	49.55	4.55	5.2	4.1	20.80
	2015	qQ15a	dm1463	7	62.12	5.38	5.3	3.9	24.10
		qQ15b	f2116	8	73.58	11.20	5.3	3.7	43.60

qVR15a, which is located in LG8, is related to vitexin-rhamnoside content and accounted for 17.70% of the variance in 2015; qV14a, qV14b, and qV14c are in LG6, LG14, and LG16, respectively; these are related to vitexin content, and in 2014, they explained 16.30–59.00% of the variance. In addition, qV15a, qV15b, qV15c, and qV15d, which are located in LG1, LG6, LG10, and LG16, respectively, are also related to vitexin content; in 2015, they explained 16.70–52.60% of the variance.

qR14a, qR14b, and qR14a, which were located in LG6, LG9, and LG15, respectively, were related to rutin content; in 2014, they explained 17.40–21.40% of the variance. qR15a, which was located in LG7, was also related to rutin content and in 2015 accounted for 25.00% of the variance. The linkage group qH14a, which is located in LG1, is related to hyperoside content and in 2014 accounted for 17.30% of the variance. qH15a, qH15b, and qH15c, which are located in LG3, LG6, and LG7, respectively, are related to hyperoside content, and in 2015, they explained 18.20–21.10% of the variance.

qQ14a, qQ14b, and qQ14c, which are located in LG1, LG3, and LG7, respectively, are related to quercetin content; in 2014, they explained 20.80–21.80% of the variance. Lastly, qQ15a and qQ15b, which are located in LG7 and LG8, respectively, are also related to quercetin content; in 2015, they explained 24.10–43.60% of the variance.

## Discussion

Genotyping with molecular markers is useful for studies on phylogeny, evolution, plant breeding, and diseases^27–29^. Restriction fragment length polymorphisms (RFLPs), randomly amplified polymorphic DNA (RAPD), amplified fragment length polymorphisms (AFLPs), and simple sequence repeats (SSRs) were once the mainstays of genotyping. However, because they are based on gel electrophoresis, these methods usually require a substantial amount of time, with high labour costs, to sample a large population. In addition, these methods are prone to error from artificial bands. Moreover, because of marker number limitations, these methods were not suitable for constructing high-density linkage maps, assembling chromosomes, constructing fine-scale QTL maps, and breeding with marker-assisted selection^30–33^.

The emergence and development of NGS technology has made RAD-seq a feasible route to genotype the entire genome in a short time. However, library construction for RAD-seq is labour-intensive and time-consuming, and thus modifications such as ddRAD^34^, SLAF^35, 36^, and 2b-RAD^23^ have been introduced. As a high-throughput sequencing technique, 2b-RAD can be used for large-scale genotyping, and compared to traditional molecular marker sequencing techniques, it can construct the linkage map with high marker density and good uniformity. The 2b-RAD method has many advantages. It can provide a streamlined alternative to existing RAD-seq library construction methods, because all reactions occur consecutively in a single well within 4 h. At the same time, 2b-RAD can detect almost every restriction site in the genome in parallel, whereas other RAD-seq methods can only detect a subset of sites. The third advantage that 2b-RAD provides is a choice of selective adaptors, which can adjust to the marker density in the genome. This choice can balance the level of genotyping detail against sequencing throughput capabilities, depending on the type of study^37^.

In this study, we constructed the refined high-density linkage map for hawthorn using the 2b-RAD method. Other studies have reported the construction of high-density linkage maps using the 2b-RAD method. Guo *et al*.^38^ found 1,385 SNP markers in rice, Tian *et al*.^39^ found 7,389 SNPs in sea cucumber, and Fu *et al*.^40^ found 3,121 SNPs in their high-density linkage map for bighead carp. Compared to linkage maps constructed using RAD and GBS methods^16, 17, 41–44^, whose marker numbers ranged from several hundreds to thousands, the linkage map in this study contained 3,894 SNP markers, comparable to and suitable for QTL mapping of flavonoid content. Moreover, these mapped markers can also be used for candidate gene discovery and de novo chromosome assembly for hawthorn.

We first conducted fine-scale QTL mapping analysis of flavonoid content for hawthorn leaves based on this high-density linkage map. Flavonoid content was measured in 2014 and 2015, and 21 QTLs related to flavonoid content were discovered in 10 linkage groups. This preliminary QTL mapping analysis for flavonoid content in hawthorn leaves will continue, especially for the QTLs that were adjacent within a single linkage group. Both qV14c and qV15d were related to vitexin content, had high LOD values (17.4 and 14.59, respectively), and explained 59.00% and 52.60% of the variance, respectively; these QTLs will be the focus of future research.

## Materials and Methods

### Plant material and DNA extraction

The diploid seed parent material came from cv. Shandongdamianqiu, the most common cultivar in Shandong Province and Beijing, China. The diploid pollen parent came from cv. Damianqiu, which is a cultivar native to Anshan city in northeast China. The parents were hybridized to produce 107 progenies (is it including direct and reciprocal cross progenies?). Genomic DNA was extracted using the CTAB method, and RNA digestion was conducted by adding the proper quantities of RNase and incubating at 37 °C for 30 min. DNA samples were checked for quality and concentration and used for further experiments.

### Flavonoid content determination

In 2014 and 2015, the content of five flavonoid monomers, vitexin-rhamnoside, vitexin, rutin, hyperoside, and quercetin, was detected using an Agilent 1100 HPLC with a DAD detector and a C18 column (250 × 4.6 mm, 5 μm). Acetonitrile (A) and 0.5% phosphoric acid solution (B) were used as the mobile phase with the following gradient elution protocol: 0–9 min, 18–20%(A); 9–25 min, 20–50%(A); 25–33 min, 50–18%(A); 33–38 min, 18%(A). The maximum absorption spectrum was 345 nm, the column temperature was 25 °C, the flow rate was 1.0 mL·min^−1^, and the injection volume was 20 μL.

### Library construction and sequencing

The 2b-RAD libraries were prepared for 109 samples by following the protocol developed by Wang *et al*.^23^. A total of 100 ng genomic DNA was digested by 4 U BsaXI (New England Biolabs) in a 15-ml reaction at 37 °C for 3 h. A 1% agarose gel was used to verify the digestion of genomic DNA (~30 ng). A total of 12 ml ligation master mix containing 0.2 mM each of two library-specific adaptors, 1 mM ATP (New England Biolabs), and 800 U T4 DNA ligase (New England Biolabs) was added to the digestion product at 4 °C for 16 h, and the mixture was inactivated by heat at 65 °C for 20 min. Ligation products were amplified in three 20 ml reactions per sample, each composed of 7 ml ligated DNA, 0.1 mM each primer, 0.3 mM dNTPs, 1× Phusion HF buffer, and 0.4 U Phusion high-fidelity DNA polymerase (New England Biolabs). PCR was conducted in a DNA Engine Tetrad 2 thermal cycler (Bio-Rad) with 20 cycles of 98 °C for 5 s, 60 °C for 20 s, and 72 °C for 10 s, with a final extension at 72 °C for 10 min. A 2% agarose gel was used for excising the target band, and the DNA will be diffused into nuclease-free water at 4 °C for 12 h. Sample-specific barcodes were introduced by PCR with platform-specific barcode-bearing primers. Each 20 ml PCR reaction contained 25 ng of gel-extracted PCR product, 0.1 mM of each primer, 0.3 mM dNTPs, 1× Phusion HF buffer, and 0.4 U Phusion high-fidelity DNA polymerase; four or five cycles of the PCR profile listed above were performed. PCR products were purified using a QIAquick PCR purification kit (Qiagen) and pooled for sequencing using the Illumina HiseqXTen platform.

### Sequence data pre-processing and de novo genotyping

Genotyping was performed using procedures described by Jiao *et al*.^37^ Raw reads were first trimmed to remove adaptor sequences. The 3′ terminal positions were excluded from each read to eliminate artefacts that might have arisen at ligation sites. Reads with no restriction sites or containing ambiguous base calls (Ns), long homopolymer regions (>10 bp), and regions with more than five consecutive low-quality (score <10) positions were removed. The remaining trimmed, high-quality reads formed the basis of subsequent analyses. De novo 2b-RAD genotyping was performed using the program RADtyping v1.0.

### Linkage map construction

lm × ll (markers from the pollen parent) or nn × np (markers from the seed parent) were categorized as the dominant markers, which segregated in a 1:1 ratio in the map population. Next, hk × hk (markers in both parents) was categorized as co-dominant markers, which were present in both parents and segregated in a ratio of 1:2:1. Before grouping, the markers were tested for goodness-of-fit based on the expected Mendelian ratios through chi-square tests to eliminate markers that significantly deviated from the expected ratios (p-value ≤ 0.05). The qualified markers were then used to construct paternal and maternal linkage maps using the JoinMap 4.1 software^26^. An LOD score cut-off of 5.0 was used to determine the genetic positions of the markers. Map distances (cM) were converted using recombination frequencies through the Kosambi mapping function. The consensus map was generated by integrating the parental maps based on the shared markers using MergeMap^44, 45^ with a map weight a 1.0. The visualized linkage maps were subsequently drawn using MapChart 2.2^46^.

### QTL mapping for content of five flavonoids

QTL mapping analysis was performed for the content of five flavonoids in hawthorn using MapQTL^47^. The LOD scores were first analysed using the interval mapping model; LOD statistics were calculated at an interval of 1 cM. Genome-wide and chromosome-wide LOD significance thresholds at the 95% level were determined with a 1000 permutation test for the content of five flavonoids, and QTLs with LOD scores greater than the LOD threshold at 95% were declared significant. Once a QTL was detected, the confidence interval was calculated using the protocol of Li^48^.

## Electronic supplementary material

Additional File A1

Additional File A2

Additional File A3

Supp Info
