# Supplementary material for: High-Density Genetic Linkage Map Construction and Quantitative Trait Locus Mapping for Hawthorn (Crataegus pinnatifida Bunge)
Source: Sci Rep. 2017 Jul 14;7:5492. doi: 10.1038/s41598-017-05756-5 (PMC5511184; doi:10.1038/s41598-017-05756-5)
Supplement: Supplementary file 4 — Supp Info [file 41598_2017_5756_MOESM4_ESM.docx]

High-Density Genetic Linkage Map Construction and Quantitative Trait Locus Mapping for Hawthorn (*Crataegus pinnatifida* Bunge)

Yuhui Zhao^1^, Kai Su^1^, Gang Wang^1^, Liping Zhang^1^, Jijun Zhang^2*^, Junpeng Li^1^, Yinshan Guo^1*^

^1^College of Horticulture, Shenyang Agricultural University, Shenyang, P.R. China

^2^College of Horticulture Sciences & Technology, Hehei Normal University of Science & Technology, China

*Corresponding author. Email: [grapeguo@yeah.net](mailto:guoyinshan77@126.com), zjjqhd@163.com














5 flavonoid component content distribution.
